# The impact of the national reimbursement drug list negotiation policy on the accessibility and utilization of evolocumab and alirocumab in different levels of hospitals: an interrupted time series analysis

**DOI:** 10.3389/fphar.2025.1612921

**Published:** 2025-09-22

**Authors:** Wan Tang, Wei Li, Anqi Zhang, Huanhuan Wu, Wei Lu, Yan Fan

**Affiliations:** ^1^ Department of Pharmacy, Nanjing Drum Tower Hospital Group Suqian Hospital, Suqian, China; ^2^ Department of Pharmacy, The Affiliated Suqian Hospital of Xuzhou Medical University, Suqian, China; ^3^ Department of Public Administration, School of Economics and Management, Suqian University, Suqian, China

**Keywords:** national reimbursement drug list negotiation, interrupted time series analysis, evolocumab, alirocumab, accessibility

## Abstract

**Objective:**

In December 2021, evolocumab and alirocumab were included in the National Reimbursement Drug List Negotiation (NRDLN), with implementation commencing in January 2022. This study aimed to evaluate the impact of the NRDLN policy on the clinical utilization of two novel lipid-lowering agents in different levels of hospitals.

**Methods:**

Data on evolocumab and alirocumab were collected from 910 tertiary and secondary public general hospitals across 31 provinces or autonomous regions in China (from January 2020 to December 2023) via the China Pharmaceutical Information Network (CPIN). Interrupted Time Series (ITS) analysis was employed to evaluate changes in drug availability, Defined Daily Doses (DDDs), and Defined Daily Dose cost (DDDc) before and after the inclusion of these drugs in the NRDLN.

**Results:**

The inclusion of evolocumab and alirocumab in the NRDLN was followed by a substantial increase in their availability, with alirocumab (β_3_ = 0.207 vs. 0.701) showing a larger improvement in availability than evolocumab. Following policy implementation, DDDs of both drugs maintained a persistent upward trend, and those in tertiary hospitals (β_3_ = 71041.810 Vs. 43625.730) consistently showed higher values than secondary hospitals (β_3_ = 3753.400 Vs. 3034.650). However, the disparity in trend slopes between hospital tiers gradually narrowed over time. A distinct breakpoint and significant trend changes in DDDc were observed for evolocumab (β_2_ = −53.587) and alirocumab (β_2_ = −79.280) after policy implementation, with DDDc values markedly lower then.

**Conclusion:**

The inclusion of evolocumab and alirocumab in the NRDLN has significantly enhanced their accessibility and eased the medication burden on patients. Furthermore, the disparity in the adoption and utilization of PCSK9 inhibitors across hospital tiers has progressively narrowed, thereby safeguarding equitable health outcomes and advancing population health security.

## Introduction

Proprotein Convertase Subtilisin/Kexin Type 9 (PCSK9) is the most potent regulator of cholesterol transport in the body. Its mechanism involves inhibiting the recycling of low-density lipoprotein receptors (LDLRs), so that levels of low-density lipoprotein (LDL) are elevated in the bloodstream. Persistent binding of LDL to cholesterol forms low-density lipoprotein cholesterol (LDL-C), thereby contributing to dyslipidemia (Hummelgaard et al., 2023).

In recent years, a novel class of lipid-lowering agents—PCSK9 inhibitors—has been approved by the European Medicines Agency (EMA) and the U.S. Food and Drug Administration (FDA). These inhibitors specifically bind to PCSK9 to block the internalization and degradation of LDLRs, thereby promoting LDL internalization and clearance to ameliorate lipid disorders (Gallego-Colon et al., 2020; Zheng et al., 2024). Extensive studies have confirmed that PCSK9 inhibitors not only effectively reduce LDL levels of the patients who fail to achieve adequate lipid control with maximally tolerated statins and ezetimibe combination therapy but also exhibit beneficial effects in anti-inflammatory responses (Qian et al., 2017; Savage et al., 2023; Zhang et al., 2023), inhibition of atherosclerotic plaque formation, and antiplatelet activity (Xiao and Su, 2022; Feng et al., 2022). As a result, PCSK9 inhibitors have emerged as a groundbreaking therapeutic strategy for lipid management, with potential implications in cardiovascular diseases and even cancer.

The National Reimbursement Drug List Negotiation (NRDLN) policy is a key mechanism implemented by the National Healthcare Security Administration (NHSA) to significantly reduce the prices of clinically essential and therapeutically proven but high-cost patented drugs and exclusive medications. Through negotiations with relevant pharmaceutical companies, the NHSA establishes medical insurance payment standards and includes these drugs in the national procurement list for basic medical insurance, work-related injury insurance, and maternity insurance (Liu et al., 2022; Mingge et al., 2023). Its implementation is critical for addressing unmet clinical needs and enhancing the cost-effectiveness of medical insurance funds. In December 2021, PCSK9 inhibitors (evolocumab and alirocumab) were formally added to the National Basic Medical Insurance, Work-Related Injury Insurance, and Maternity Insurance Drug List (2021 Edition) under this policy (National Healthcare Security Administration and National Health Commission, 2021). Interrupted Time Series (ITS) analysis, a quasi-experimental methodology, evaluates policy interventions by comparing pre- and post-intervention trends and level changes in observed indicators across multiple time points (Bernal et al., 2017). Currently, this method has been recognized as the most robust quasi-experimental approach for evaluating the longitudinal effects of policy interventions (Hudson et al., 2019; Wagner et al., 2002). This study employed ITS to analyze shifts in accessibility and utilization metrics for evolocumab and alirocumab before and after their inclusion in the NRDLN. The findings aim to elucidate the effects of the NRDLN policy on PCSK9 inhibitors’ accessibility and clinical adoption, thereby providing evidence-based insights for optimizing national drug reimbursement strategies.

## Materials and methods

### Sample and data source

The data for this study were sourced from the China Pharmaceutical Information Network (CPIN) (https://www.cpi.ac.cn/index.html), encompassing 910 public general hospitals (including 311 secondary hospitals and 599 tertiary hospitals) across 31 provinces or autonomous regions in China. With January 2022—the official implementation time of the inclusion of evolocumab and alirocumab in the NRDLN—selected as the intervention point, monthly data were collected for both pre-intervention (from January 2020 to December 2021) and post-intervention (from January 2022 to December 2023) periods. Key variables included generic drug names, manufacturers, dosage forms, strengths, procurement volume, and procurement expenditure.

### Measurements

The NRDLN has improved medication availability through measures such as the “Dual-channel” policy for drugs, hospital performance metrics tied to out-of-pocket payment ratios under basic insurance, and incentive policies for innovative drugs—thereby potentially increasing their Defined Daily Doses (DDDs, measuring usage frequency). Simultaneously, through forceful price negotiations, it has driven a direct and significant reduction in the Defined Daily Dose cost (DDDc, daily medication cost) (Fu and Mao, 2023). Furthermore, the defined reimbursement scope and reforms in medical insurance payment methods have exerted moderating and consolidating effects on both DDDs and DDDc. Therefore, in line with the WHO/HAI methodology and informed by previous domestic and international studies (Chen et al., 2022; Asmamaw et al., 2024; Cheng et al.), this study evaluated the accessibility of evolocumab and alirocumab from three dimensions: availability, DDDs and DDDc. Data analysis was conducted through Microsoft Excel, covering the collection period from January 2021 to December 2023.

### Availability

We defined availability as the proportion of all the surveyed hospitals that stocked the investigated drug. Furthermore, we compared the availability of these drugs between tertiary and secondary hospitals before and after policy implementation.

$$\text{Availability}=\frac{\text{number}\;\text{of}\;\text{helthcare}\;\text{facilities}\;\text{stocking}\;\text{the}\;\text{drug}}{\text{total}\;\text{surveyed}\;\text{facilities}}\times 100\%$$

The availability levels of medicines can be classified as follows according to WHO standards (WHO, 2006; Fang et al., 2021): availability: <30%, means that the medicine had very low availability in surveyed hospitals; availability: 30%–49%, means that the medicine had low availability in surveyed hospitals; availability: 50%–80%, means that the medicine was available in many surveyed hospitals; availability: >80%, means that the medicine had good availability and was available in most surveyed hospitals.

### Cost

We used DDDc to reflect the cost and their financial burden on patients. DDDc represents the average daily treatment cost for a patient who uses the drug, and a higher DDDc value indicates a heavier economic burden (Wu et al., 2023; Gao et al., 2021), which is calculated based on the DDDs. DDDs are calculated as the annual drug consumption divided by the DDD value, with the DDD value primarily sourced from the WHO Collaborating Centre for Drug Statistics Methodology. If a DDD value is not listed by WHO, it will be determined based on drug specifications or authoritative references such as the Newly Compiled Pharmacology (18th Edition) (Chen et al., 2019). Notably, higher DDDs reflect greater clinical utilization intensity and preference for the drug (Ezzat et al., 2024).

### Statistical analysis

In this study, the monthly availability rate, DDDs, and DDDc of evolocumab and alirocumab were used as outcome measures (Supplementary Tables S1 and S2). ITS model was constructed as follows (Ezzat et al., 2024; Schaffer et al., 2021; Yang et al., 2021; Nakakande et al., 2024):

$$Y_{t}=\beta_{0}+\beta_{1}\times T_{1}+\beta_{2}\times T_{2}+\beta_{3}\times T_{3}+\varepsilon$$

Where *Y*_*t*_ is the observed outcome metrics at each t time point (monthly availability, DDDs and DDDc). T_1_ refers to a continuous variable indicating the number of months at time t from the beginning of the observation period (from January 2020 to December 2023, T_1_ = 1, 2, 3 … 48). T_2_ is the dummy variable for the two time periods before and after policy implementation (T_2_ *=* 0 represents the time period before policy implementation, namely, from January 2020 to December 2021, and T_2_ *=* 1 represents the time period after policy implementation, namely, from January 2022 to December 2023). T_3_ is the time point after policy intervention (T_3_ = 0 indicates the time before policy intervention, and T_3_ = 1, 2, 3 … 24 indicates the month(s) after policy intervention. ε is the error term. β_0_ is the intercept (which refers to the outcome when the time is 0 at the baseline level), β_1_ reflects the baseline trend during the pre-intervention period, β_2_ is the level of changes in the intervention (difference between the last pre-policy and first post-policy observations), β_3_ is the trend change of the outcome caused by the policy intervention (slope deviation from the baseline). When conducting ITS analysis, autocorrelation in the data might bias the results. Therefore, the Cumby-Huizinga test was employed to assess the presence of autocorrelation. All analyses were performed through STATA v.17 software (StataCorp LLC, Texas, United States).

## Results

### Changes in availability

**FIGURE 1:**
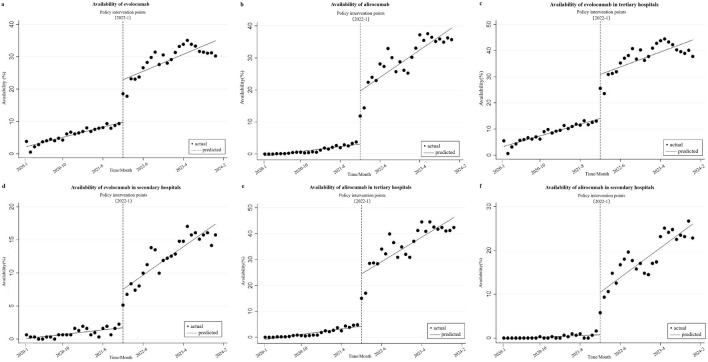
Results of the regression analysis of the availability of study drugs before and after policy implementation shown in a scatter plot. **(a)** Availability of evolocumab, **(b)** Availability of alirocumab, **(c)** Availability of evolocumab in tertiary hospitals, **(d)** Availability of evolocumab in secondary hospitals, **(e)** Availability of alirocumab in tertiary hospitals, **(f)** Availability of alirocumab in secondary hospitals.

Figure 1 and Table 1 show the changes of the availability of evolocumab and alirocumab. As shown in Figure 1a, the availability of evolocumab exhibited a slow upward trend before policy implementation (β_1_ = 0.317, *P* < 0.001); when it was included in the NRDLN, its availability saw a notable increase (β_2_ = 13.031, *P* < 0.001); after its inclusion, the overall upward trend in its availability persisted but showed no statistically significant difference compared with the pre-policy trend (β_3_ = 0.207, *P =* 0.118). As shown in Figure 1b, the availability of alirocumab showed a gradual increase before policy implementation (β_1_ = 0.154, *P* < 0.001). After its entry into the NRDLN, the availability of alirocumab significant increase (β_2_ = 16.593, *P* < 0.001). Policy intervention resulted in a statistically significant upward trend in its availability (β_3_ = 0.701, *P* < 0.001). Which suggests that the NRDLN policy was associated with a larger availability improvement for alirocumab than for evolocumab. Figures 1c–f show the trends in availability of evolocumab and alirocumab in secondary and tertiary hospitals before and after their inclusion in the NRDLN, which is basically consistent with the trend of their overall availability. Compared with the pre-policy period, the difference in slopes of availability between secondary and tertiary hospitals for evolocumab and alirocumab narrowed after policy implementation (Tertiary hospitals, β_3_ = 0.126, *P* = 0.446 vs. β_3_ = 0.733, *P* < 0.001; Secondary hospitals, β_3_ = 0.362, *P* < 0.001 vs. β_3_ = 0.678, *P* < 0.001). This demonstrates that the implementation of the NRDLN policy has facilitated the clinical adoption of evolocumab and alirocumab, alongside improved availability of these PCSK9 inhibitors.

**TABLE 1:** Changes in levels and trends of availability, DDDs and DDDc.

	Evolocumab			Alirocumab		
	Coefficient	*P* value	95% CI	Coefficient	*P* value	95% CI
Availability (%)						
Baseline line (β_0_)	2.240	<0.001	1.497–2.982	−0.615	0.004	−1.028 to −0.202
Baseline trend (β_1_)	0.317	<0.001	0.268–0.367	0.154	<0.001	0.122–0.186
Level change immediately after intervention (β_2_)	13.031	<0.001	9.688–16.375	16.593	<0.001	12.018–21.168
Trend change after intervention (β_3_)	0.207	<0.001	−0.053–0.466	0.701	<0.001	0.409–0.992
Tertiary hospitals						
Baseline line (β_0_)	3.356	<0.001	2.226–4.486	−0.839	0.003	−1.398 to −0.280
Baseline trend (β_1_)	0.447	<0.001	0.373–0.520	0.214	<0.001	0.171–0.257
Level change immediately after intervention (β_2_)	16.786	<0.001	12.447–21.124	20.188	<0.001	14.578–25.798
Trend change after intervention (β_3_)	0.126	0.466	−0.213–0.465	0.733	<0.001	0.367–1.099
Secondary hospitals						
Baseline line (β_0_)	0.091	0.580	−0.231–0.413	−0.182	0.084	−0.389–0.024
Baseline trend (β_1_)	0.068	<0.001	0.039–0.096	0.039	0.003	0.013–0.065
Level change immediately after intervention (β_2_)	5.799	<0.001	4.101–7.498	9.669	<0.001	6.916–12.422
Trend change after intervention (β_3_)	0.362	<0.001	0.242–0.482	0.639	<0.001	0.474–0.803
DDDs						
Baseline line (β_0_)	4262.020	0.115	−1039.870–9563.910	−2683.102	0.016	−4866.953 to −499.251
Baseline trend (β_1_)	2931.563	<0.001	2453.214–3409.913	833.907	<0.001	587.608–1080.206
Level change immediately after intervention (β_2_)	358427.900	<0.001	232729.700–484126.200	24277.340	0.392	−31268.960 to 79823.650
Trend change after intervention (β_3_)	74795.210	<0.001	63964.010–85626.420	46660.370	<0.001	43057.310–50263.440
Tertiary hospitals						
Baseline line (β_0_)	4323.620	0.110	−976.642–9623.882	−2639.259	0.017	−4806.041 to −472.478
Baseline trend (β_1_)	2904.598	<0.001	2424.604–3384.502	825.012	<0.001	579.322–1071.702
Level change immediately after intervention (β_2_)	365058.700	<0.001	236118.200–493999.200	24228.410	0.365	−28149.180 to 76606.000
Trend change after intervention (β_3_)	71041.810	<0.001	60012.020–82071.610	43625.730	<0.001	40245.390–47006.060
Secondary hospitals						
Baseline line (β_0_)	−61.600	0.161	−147.700–24.500	−43.843	0.052	−88.115–0.430
Baseline trend (β_1_)	26.965	<0.001	19.517–34.413	8.895	<0.001	4.284–13.506
Level change immediately after intervention (β_2_)	−6630.765	0.078	−14017.02–755.494	48.929	0.978	−3504.428–3602.287
Trend change after intervention (β_3_)	3753.400	<0.001	3280.144–4226.656	3034.650	<0.001	2736.776–3332.524
DDDc (CNY)						
Baseline line (β_0_)	99.741	<0.001	93.829–105.652	96.732	0.007	26.252–167.213
Baseline trend (β_1_)	−1.083	0.001	−1.696–0.471	0.176	0.940	−4.403–4.755
Level change immediately after intervention (β_2_)	−53.587	<0.001	−63.746 to −43.428	−79.280	0.001	−127.234 to −31.324
Trend change after intervention (β_3_)	1.090	0.001	0.474–1.707	−0.154	0.948	−4.736–4.429

DDDs, Defined Daily Doses; DDDc, Defined Daily Dose cost; CNY, China Yuan; CI, confidence interval.

### Changes in DDDs

**FIGURE 2:**
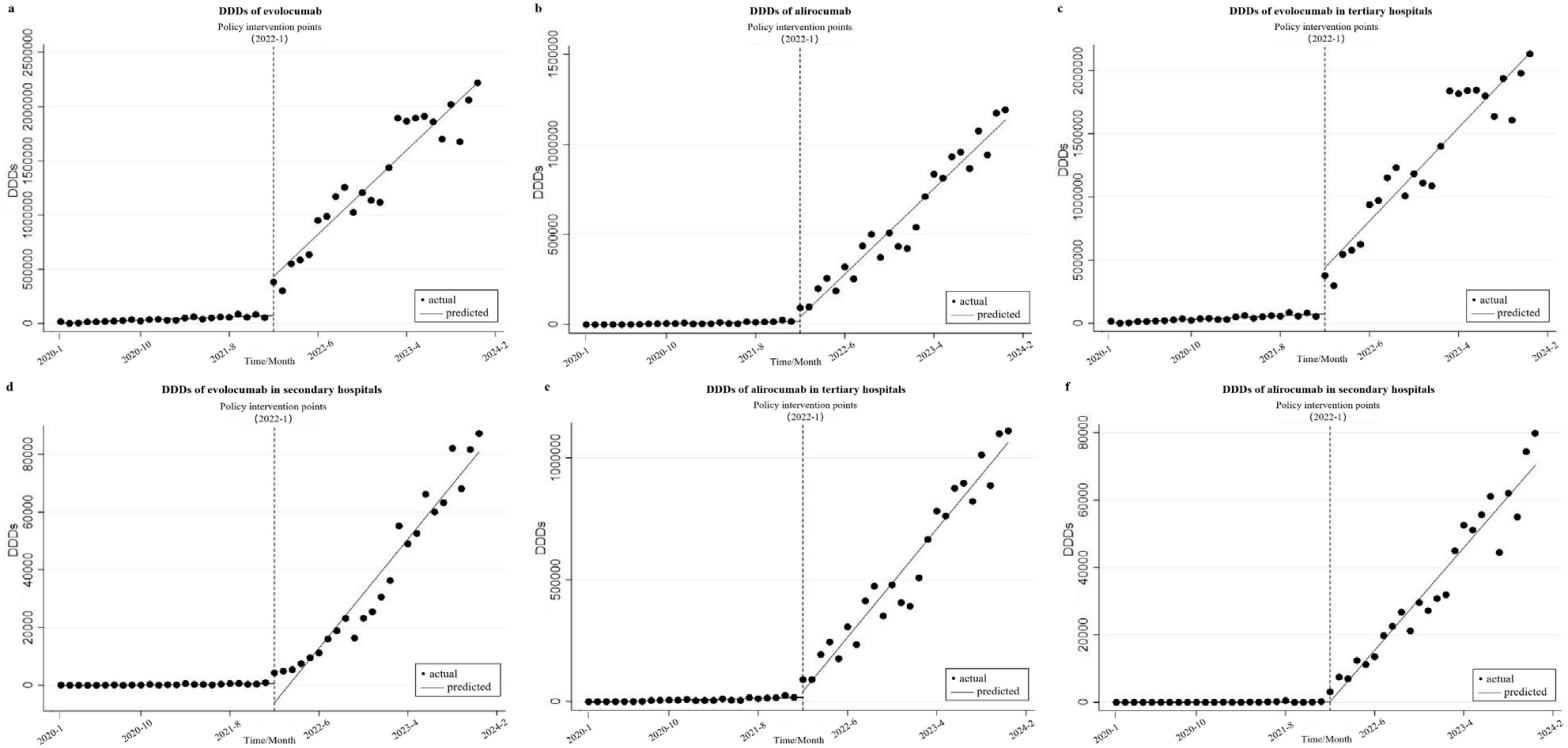
Results of the regression analysis of the Defined Daily Doses (DDDs) of study drugs before and after policy implementation shown in a scatter plot. **(a)** DDDs of evolocumab, **(b)** DDDs of alirocumab, **(c)** DDDs of evolocumab in tertiary hospitals, **(d)** DDDs of evolocumab in secondary hospitals, **(e)** DDDs of alirocumab in tertiary hospitals, **(f)** DDDs of alirocumab in secondary hospitals.

Figures 2a,b show that before policy implementation, evolocumab exhibited an upward trend in DDDs (β_1_ = 2931.563, *P* < 0.001), while alirocumab also demonstrated an increasing trend (β_1_ = 833.907, *P* < 0.001), albeit less pronounced than that of evolocumab. In the month of its inclusion in the NRDLN, evolocumab showed a substantial increase in total DDDs compared with the preceding month, with a statistically significant immediate level change (β_2_ = 358427.900, *P* < 0.001). Alirocumab also showed a marked increase in total DDDs, but its immediate level change lacked statistical significance (β_2_ = 24277.340, *P* = 0.392). Following policy implementation, both drugs continued an upward trend in total DDDs, with statistically significant differences (β_3_ = 74795.210, *P* < 0.001 vs. β_3_ = 46660.370, *P* < 0.001). However, the trend difference between the two drugs narrowed compared with that in the pre-policy period. Figures 2c–f show the disparities in DDDs changes of the investigated drugs between secondary and tertiary hospitals. The study found that the increase in DDDs was significantly greater in tertiary hospitals compared with secondary hospitals (Evolocumab, β_3_ = 71041.810,*P* < 0.001 vs. β_3_ = 3753.400,*P* < 0.001; Alirocumab, β_3_ = 43625.730, *P* < 0.001 vs. β_3_ = 3034.650, *P* < 0.001), which aligned with hospital tier disparities in resource allocation and clinical capacity. In the month of policy implementation, evolocumab showed a non-significant decline in DDDs in secondary hospitals (β_2_ = −6630.765, *P* = 0.078). Following policy implementation, both evolocumab and alirocumab exhibited statistically significant and basically the same upward trends in DDDs in secondary hospitals (β3 = 43625.730, *P* < 0.001 vs. β3 = 3034.650, *P* < 0.001). However, the magnitude of utilization growth remained more pronounced for evolocumab than for alirocumab.

### Changes in DDDc

**FIGURE 3:**
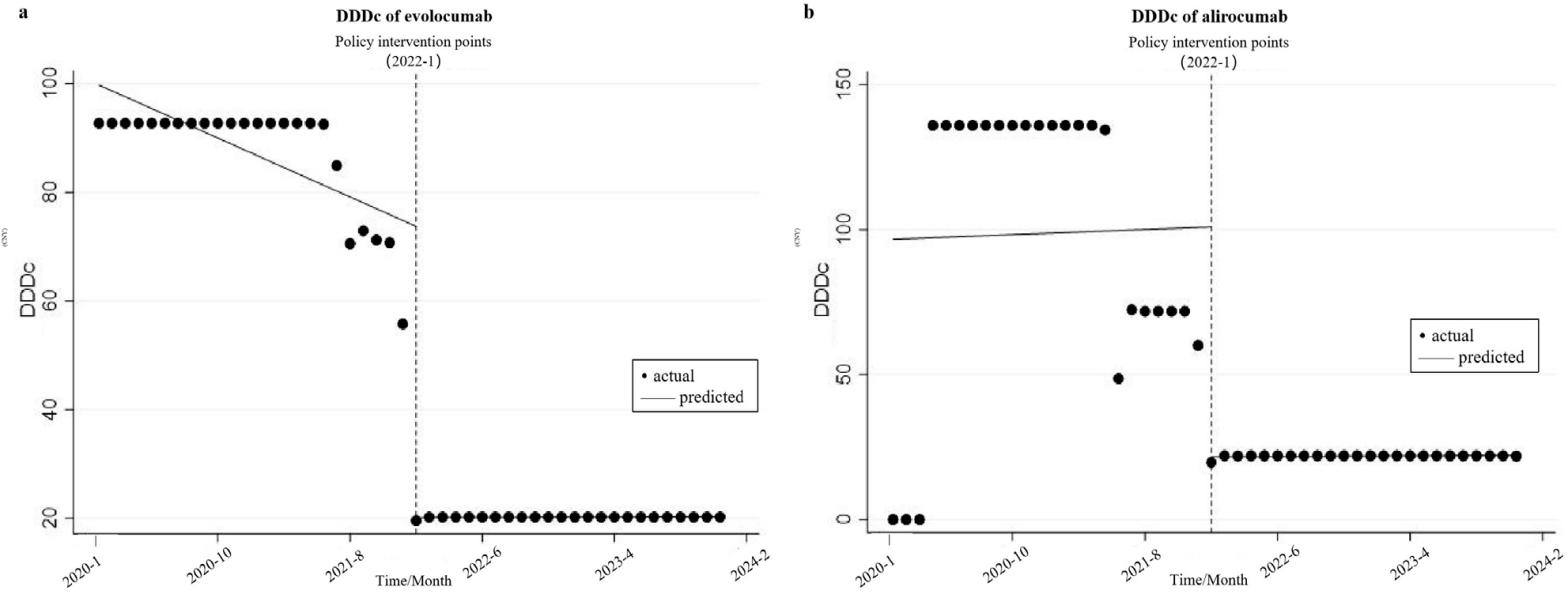
Results of the regression analysis of the Defined Daily Doses Cost (DDDc) of study drugs before and after policy implementation shown in a scatter plot. **(a)** DDDc of evolocumab, **(b)** DDDc of alirocumab.

Figure 3 shows the changes in DDDc of evolocumab and alirocumab before and after the implementation of the NRDLN policy, and Table 1 shows the ITS results. Statistical results show that before the implementation of the policy, the DDDc of evolocumab exhibited a statistically significant downward trend (β_1_ = −1.083, *P* = 0.001), but that of alirocumab displayed a gradual upward trend without statistical significance (β_1_ = 0.176, *P* = 0.940). During the month of policy implementation, a reduction in DDDc was observed for both evolocumab and alirocumab, and the change was statistically significant (β_2_ = −53.587, *P* < 0.001 vs. β_2_ = −79.280, *P* = 0.001). Compared to the pre-intervention trend, evolocumab exhibited a slight but statistically significant increase in the DDDc trend (β_3_ = 1.090, *P* = 0.001), while alirocumab continued to show a declining trend that was not statistically significant (β_3_ = −0.154, *P* = 0.948). The implementation of the NRDLN policy has accelerated the downward trend in the costs of evolocumab and alirocumab, effectively alleviating the medication burden on patients.

## Discussion

According to the China Cardiovascular Health and Disease Report 2024 (National Center for Cardiovascular Diseases, 2025), the number of incident ASCVD cases has increased from 5.3007 million in 1990 to 12.3411 million, representing a rise of 132.82%. Meanwhile, the mortality rate rose from 204.78 per 100,000 to 322.30 per 100,000, an increase of 57.39%. The ESC/EAS Dyslipidemia Guidelines recommend an LDL-C target of ≤1.4 mmol/L for patients with ASCVD at very high risk (Mach et al., 2020). However, in China, less than 10% of patients achieve this LDL-C target (Liu et al., 2021). Real-world studies have demonstrated that treatment with PCSK9 inhibitors significantly reduces LDL-C levels and improves clinical outcomes, with no significant adverse effects (Liu et al., 2021; Gargiulo et al., 2023; Gargiulo et al., 2024). Nevertheless, their high cost has historically hindered their widespread adoption. The limited access to this novel therapy for patients resulted in the low adoption rate of PCSK9 inhibitors in China (Weintraub and Gidding, 2016). This study utilized procurement data of evolocumab and alirocumab from representative hospitals nationwide and applied an ITS analysis to investigate changes in accessibility and clinical utilization of the two PCSK9 inhibitors before and after their inclusion in the NRDLN. As anticipated, the implementation of the NRDLN policy has enhanced the accessibility of evolocumab and alirocumab, thereby effectively alleviating the medication burden on patients. These findings further confirm the positive impact of the policy on improving access to innovative PCSK9 inhibitors, with both short-term and long-term changes observed.

### Impact of the NRDLN on availability

The study found that the availability of evolocumab and alirocumab demonstrated an immediate increase following their inclusion in the NRDLN, with a subsequent amplified rise. However, their overall availability remained lower (<50%) (WHO, 2006). By the end of 2023, the overall availability of evolocumab and alirocumab stood at merely 30.22% and 35.71%, respectively, a level that remained suboptimal.

By the end of 2023, the availability rates of evolocumab and alirocumab in tertiary hospitals were 37.73% and 42.40%, while in secondary hospitals, the rates were 15.76% and 22.83%. These figures indicate that the availability of these drugs remained suboptimal, which might be attributed to the relatively late market entry of evolocumab and alirocumab in China, coupled with limited clinical experience among physicians regarding these agents. Consequently, clinicians tended to favor prescribing therapeutically familiar medications with established usage histories (Liu et al., 2023). On the other hand, although evolocumab and alirocumab were included in the NRDLN, their prices remained significantly higher than those of traditional lipid-lowering medications. Against the backdrop of diagnosis-related groups (DRGs)/diagnosis-intervention packet (DIP) payment system reforms (National Healthcare Security Administration, 2024) and the implementation of policies such as zero markup drug pricing (Wang et al., 2021), healthcare institutions tended to prioritize cost-effective alternatives to control treatment expenditures. These factors have collectively affected the broader adoption of PCSK9 inhibitors, resulting in their suboptimal availability. Furthermore, our analysis revealed that while the availability of evolocumab in secondary hospitals remained lower than in tertiary hospitals (in December 2023, 15.76% VS 37.73%), secondary hospitals exhibited a steeper slope of increase in availability over time. Alirocumab demonstrated lower availability rates in secondary hospitals than in tertiary hospitals (in December 2023, 22.83% vs. 42.4%). However, the availability of alirocumab showed significantly steeper growth slopes relative to that of evolocumab in both secondary and tertiary hospitals. In summary, these findings demonstrate that the availability of alirocumab and secondary hospitals exhibited stronger responsiveness to the NRDLN policy, which possibly resulted from its lower baseline availability or hospital tier disparities. Meanwhile, the policy implementation has progressively narrowed the availability gap between evolocumab and alirocumab across hospital tiers, promoting equity in drug availability.

### Impact of the NRDLN on DDDs

Our analysis revealed that following the implementation of the NRDLN policy, the DDDs of both evolocumab and alirocumab experienced an immediate surge, accompanied by a sustained upward trajectory in their growth trends. The DDDs of evolocumab and alirocumab in secondary hospitals were consistently lower than those in tertiary hospitals, and their growth slopes in secondary hospitals were also less steep than that in tertiary hospitals. These disparities align with the hierarchical structure of China’s hospital system, reflecting tier-specific variations in clinical adoption patterns. As of December 2023, the DDDs of alirocumab remained lower than those of evolocumab (1190639 vs. 2219210). This disparity may stem from the fact that alirocumab (on 28 December 2019) received CFDA approval significantly later than evolocumab (on 30 July 2018). Consequently, clinicians and patients exhibit substantially higher awareness and acceptance of evolocumab than alirocumab, leading to a prescribing preference for the former. However, following the implementation of the NRDLN policy, the disparity in growth slopes between evolocumab and alirocumab has progressively narrowed. This finding further corroborates that the policy has accelerated the adoption of PCSK9 inhibitors, enabling healthcare institutions and the general public to integrate high-cost innovative therapies into clinical practice more rapidly.

### Impact of the NRDLN on DDDc

Following their inclusion in the NRDLN, evolocumab and alirocumab demonstrated a significant reduction in DDDc. However, this downward trend was not sustained indefinitely; and the DDDc eventually plateaued at a stabilized level after reaching a specific threshold. The steepest decline in DDDc occurred in the implementation month of the NRDLN policy (namely, in January 2022), with alirocumab exhibiting a significantly steeper reduction in DDDc slope compared with evolocumab. This discrepancy may be attributable to the fact that alirocumab market pricing (1888.0 CNY) was substantially higher than evolocumab (1298.0 CNY) before their inclusion in the NRDLN. Notably, both drugs exhibited slight upward slopes in DDDc after policy implementation. This is attributed to a floor effect, potentially due to the artificially suppressed pricing of evolocumab and alirocumab during the initial enforcement phase of their entry into the NRDLN (in January 2022). It may also be linked to the volume-price agreements entered by the National Healthcare Security Administration with manufacturers during the medical insurance and price negotiation process for drug procurement (Ding et al., 2020). From January 2020 to December 2023, the DDDc of evolocumab decreased from 92.71 CNY to 20.27 CNY, and that of alirocumab dropped from 135.94 CNY to 21.89 CNY, because of the implementation of the NRDLN policy. In contrast to the findings on availability and DDDs, the changes in DDDc showed no significant disparities across hospital tiers (secondary vs. tertiary). This serves as critical evidence that the negotiated prices for evolocumab and alirocumab under the NRDLN policy were equitably adopted nationwide, regardless of hospital hierarchy, thereby significantly alleviating the financial burden on patients.

In recent years, China has faced escalating pressure on its healthcare system due to a rapidly aging population and a surging prevalence of cardiovascular and cerebrovascular chronic diseases. These demographic and epidemiological shifts have triggered excessive growth in medical insurance fund expenditures, thereby intensifying the financial strain on public healthcare coverage. To address this challenge, reforms such as the zero-markup policy on drugs and the implementation of DRG/DIP payment systems have been introduced. These measures have compelled healthcare institutions to urgently optimize cost containment strategies while maintaining service quality. Studies have confirmed that the implementation of the NRDLN policy has brought substantial reductions in drug prices, not only alleviating the financial burden on patients but also easing the financial pressure on healthcare institutions. Furthermore, this policy has improved the adoption and accessibility of innovative drugs, thereby enhancing equitable access to advanced therapies and safeguarding public health outcomes. This study primarily focused on comparing the accessibility and utilization data of evolocumab and alirocumab in secondary and tertiary public general hospitals before and after their inclusion in the NRDLN. The analysis featured comprehensive coverage and a robust comparative framework, highlighting the policy longitudinal impact. Furthermore, by conducting a comparative analysis of PCSK9 inhibitor utilization between secondary and tertiary hospitals, this study provided direct evidence that the implementation of the NRDLN policy has narrowed disparities in the adoption of innovative therapies across healthcare tiers. This convergence ensures that patients can access advanced treatments more equitably, thereby enabling the public to receive better diagnostic and therapeutic healthcare services. This study also has some limitations. Although ITS is a powerful quasi-experimental tool that can effectively reduce statistical bias, unmeasured confounders and concurrent policies (e.g., DRG/DIP reforms, local procurement initiatives), it may still affect the results. Furthermore, underreporting of hospital procurement data or missing private hospital data may have an impact on the data in this study. Subsequent research can, therefore, refine PCSK9 inhibitor data collection and investigate additional influencing factors, providing a more objective and authentic assessment of their accessibility and utilization.

## Conclusion

The study demonstrated that the NRDLN policy has promoted the adoption of innovative lipid-lowering agents (PCSK9 inhibitors) and effectively reduced disparities in their accessibility and utilization across different tiers of healthcare institutions. Secondary hospitals experienced a dramatic improvement in the accessibility of PCSK9 inhibitors after their inclusion in the NRDLN, significantly narrowing the accessibility gap with tertiary hospitals. However, a persistent divide remains between the current status and the ultimate goal of maximizing the accessibility of these drugs. Concerted efforts are required to enhance policy implementation and amplify its impact on public health outcomes.

## Data Availability

The datasets presented in this study can be found in online repositories. The names of the repository/repositories and accession number(s) can be found in the article/Supplementary Material.
