# Supplementary material for: The impact of the national reimbursement drug list negotiation policy on the accessibility and utilization of evolocumab and alirocumab in different levels of hospitals: an interrupted time series analysis
Source: Front Pharmacol. 2025 Sep 22;16:1612921. doi: 10.3389/fphar.2025.1612921 (PMC12497621; doi:10.3389/fphar.2025.1612921)
Supplement: Supplementary file 1 [file Table1.docx]

**Supplementary Table 1. Availability, DDDs and DDDc of Evolocumab from January 2020 to December 2023**

| Year | Month | DDD | Availability | DDDs | DDDc (CNY) | Tertiary hospital | | Secondary hospital | |
| --- | --- | --- | --- | --- | --- | --- | --- | --- | --- |
|  |  |  |  |  |  | Availability | DDDs | Availability | DDDs |
| 2020 | 1 | 10mg | 3.85 | 16730 | 92.71 | 5.51 | 16632 | 0.64 | 98 |
| 2020 | 2 |  | 0.55 | 392 | 92.71 | 0.67 | 364 | 0.32 | 28 |
| 2020 | 3 |  | 2.20 | 3934 | 92.71 | 3.17 | 3906 | 0.32 | 28 |
| 2020 | 4 |  | 2.86 | 13958 | 92.71 | 4.34 | 13958 |  |  |
| 2020 | 5 |  | 3.74 | 14434 | 92.71 | 5.68 | 14434 |  |  |
| 2020 | 6 |  | 4.07 | 18158 | 92.71 | 6.01 | 18088 | 0.32 | 70 |
| 2020 | 7 |  | 4.51 | 21364 | 92.71 | 6.68 | 21196 | 0.32 | 168 |
| 2020 | 8 |  | 4.07 | 27258 | 92.71 | 6.18 | 27258 |  |  |
| 2020 | 9 |  | 4.84 | 35924 | 92.71 | 7.01 | 35798 | 0.64 | 126 |
| 2020 | 10 |  | 4.29 | 23184 | 92.71 | 6.18 | 23072 | 0.64 | 112 |
| 2020 | 11 |  | 6.15 | 36470 | 92.71 | 9.02 | 36162 | 0.64 | 308 |
| 2020 | 12 |  | 6.70 | 39298 | 92.71 | 9.85 | 39242 | 0.64 | 56 |
| 2021 | 1 |  | 6.15 | 29484 | 92.71 | 8.51 | 29274 | 1.61 | 210 |
| 2021 | 2 |  | 6.48 | 29414 | 92.71 | 9.18 | 29232 | 1.29 | 182 |
| 2021 | 3 |  | 6.92 | 50876 | 92.71 | 9.52 | 50288 | 1.93 | 588 |
| 2021 | 4 |  | 8.02 | 61810 | 92.71 | 11.35 | 61516 | 1.61 | 294 |
| 2021 | 5 |  | 6.92 | 40096 | 92.71 | 10.18 | 39816 | 0.64 | 280 |
| 2021 | 6 |  | 7.58 | 51842 | 92.54 | 11.02 | 51688 | 0.96 | 154 |
| 2021 | 7 |  | 7.91 | 60354 | 84.93 | 11.85 | 59976 | 0.32 | 378 |
| 2021 | 8 |  | 8.13 | 56728 | 70.56 | 11.52 | 56140 | 1.61 | 588 |
| 2021 | 9 |  | 9.34 | 85624 | 72.94 | 13.19 | 84980 | 1.93 | 644 |
| 2021 | 10 |  | 7.91 | 57078 | 71.22 | 11.69 | 56770 | 0.64 | 308 |
| 2021 | 11 |  | 8.79 | 82824 | 70.74 | 12.52 | 82404 | 1.61 | 420 |
| 2021 | 12 |  | 9.34 | 54166 | 55.78 | 13.02 | 53242 | 2.25 | 924 |
| 2022 | 1 |  | 18.57 | 382060 | 19.58 | 25.54 | 377762 | 5.14 | 4298 |
| 2022 | 2 |  | 17.80 | 301938 | 20.25 | 23.54 | 297038 | 6.75 | 4900 |
| 2022 | 3 |  | 23.19 | 550578 | 20.27 | 30.88 | 545188 | 8.36 | 5390 |
| 2022 | 4 |  | 23.08 | 585298 | 20.27 | 31.22 | 577808 | 7.40 | 7490 |
| 2022 | 5 |  | 23.74 | 634578 | 20.27 | 31.89 | 625044 | 8.04 | 9534 |
| 2022 | 6 |  | 26.59 | 950908 | 20.27 | 35.23 | 939680 | 9.97 | 11228 |
| 2022 | 7 |  | 28.24 | 987686 | 20.27 | 37.06 | 971656 | 11.25 | 16030 |
| 2022 | 8 |  | 29.78 | 1170526 | 20.27 | 38.06 | 1151668 | 13.83 | 18858 |
| 2022 | 9 |  | 31.43 | 1255044 | 20.27 | 40.73 | 1231860 | 13.50 | 23184 |
| 2022 | 10 |  | 27.58 | 1024310 | 20.27 | 36.73 | 1007930 | 9.97 | 16380 |
| 2022 | 11 |  | 30.55 | 1206660 | 20.27 | 40.23 | 1183420 | 11.90 | 23240 |
| 2022 | 12 |  | 28.02 | 1136646 | 20.27 | 36.23 | 1111180 | 12.22 | 25466 |
| 2023 | 1 |  | 29.12 | 1117858 | 20.27 | 37.73 | 1087254 | 12.54 | 30604 |
| 2023 | 2 |  | 31.32 | 1436890 | 20.27 | 40.90 | 1400602 | 12.86 | 36288 |
| 2023 | 3 |  | 33.19 | 1894256 | 20.27 | 42.74 | 1839054 | 14.79 | 55202 |
| 2023 | 4 |  | 33.85 | 1866564 | 20.27 | 43.74 | 1817578 | 14.79 | 48986 |
| 2023 | 5 |  | 35.05 | 1894746 | 20.27 | 44.41 | 1842106 | 17.04 | 52640 |
| 2023 | 6 |  | 33.85 | 1911826 | 20.27 | 43.24 | 1845648 | 15.76 | 66178 |
| 2023 | 7 |  | 33.30 | 1860040 | 20.27 | 42.24 | 1800036 | 16.08 | 60004 |
| 2023 | 8 |  | 31.65 | 1700524 | 20.27 | 40.23 | 1637272 | 15.11 | 63252 |
| 2023 | 9 |  | 31.43 | 2020382 | 20.27 | 39.57 | 1938272 | 15.76 | 82110 |
| 2023 | 10 |  | 31.10 | 1676276 | 20.27 | 38.90 | 1608152 | 16.08 | 68124 |
| 2023 | 11 |  | 31.21 | 2060926 | 20.27 | 40.07 | 1979250 | 14.15 | 81676 |
| 2023 | 12 |  | 30.22 | 2219210 | 20.27 | 37.73 | 2131976 | 15.76 | 87234 |

Note: CNY: China Yuan
