# Supplementary material for: The impact of the national reimbursement drug list negotiation policy on the accessibility and utilization of evolocumab and alirocumab in different levels of hospitals: an interrupted time series analysis
Source: Front Pharmacol. 2025 Sep 22;16:1612921. doi: 10.3389/fphar.2025.1612921 (PMC12497621; doi:10.3389/fphar.2025.1612921)
Supplement: Supplementary file 2 [file Table2.docx]

**Supplementary Table 2. Availability, DDDs and DDDc of Alirocumab from January 2020 to December 2023**

| Year | Month | DDD | Availability | DDDs | DDDc (CNY) | Tertiary hospital | | Secondary hospital | |
| --- | --- | --- | --- | --- | --- | --- | --- | --- | --- |
|  |  |  |  |  |  | Availability | DDDs | Availability | DDDs |
| 2020 | 1 | 5.4mg |  |  |  |  |  |  |  |
| 2020 | 2 |  |  |  |  |  |  |  |  |
| 2020 | 3 |  |  |  |  |  |  |  |  |
| 2020 | 4 |  | 0.11 | 347 | 135.94 | 0.17 | 347 |  |  |
| 2020 | 5 |  | 0.11 | 139 | 135.94 | 0.17 | 139 |  |  |
| 2020 | 6 |  | 0.11 | 278 | 135.94 | 0.17 | 278 |  |  |
| 2020 | 7 |  | 0.22 | 1264 | 135.94 | 0.33 | 1264 |  |  |
| 2020 | 8 |  | 0.44 | 4333 | 135.94 | 0.67 | 4333 |  |  |
| 2020 | 9 |  | 0.55 | 4861 | 135.94 | 0.83 | 4861 |  |  |
| 2020 | 10 |  | 0.55 | 5958 | 135.94 | 0.67 | 5944 | 0.32 | 14 |
| 2020 | 11 |  | 0.33 | 5514 | 135.94 | 0.50 | 5514 |  |  |
| 2020 | 12 |  | 0.44 | 9139 | 135.94 | 0.67 | 9139 |  |  |
| 2021 | 1 |  | 0.66 | 3528 | 135.94 | 0.83 | 3472 | 0.32 | 56 |
| 2021 | 2 |  | 0.55 | 4097 | 135.94 | 0.83 | 4097 |  |  |
| 2021 | 3 |  | 1.21 | 4403 | 136.01 | 1.84 | 4403 |  |  |
| 2021 | 4 |  | 1.87 | 11347 | 135.94 | 2.50 | 11250 | 0.64 | 97 |
| 2021 | 5 |  | 1.54 | 5444 | 134.42 | 2.17 | 5403 | 0.32 | 42 |
| 2021 | 6 |  | 2.09 | 4597 | 48.60 | 2.67 | 4458 | 0.96 | 139 |
| 2021 | 7 |  | 2.64 | 16014 | 72.36 | 3.67 | 15792 | 0.64 | 222 |
| 2021 | 8 |  | 1.98 | 12389 | 71.86 | 2.50 | 11889 | 0.96 | 500 |
| 2021 | 9 |  | 2.86 | 14153 | 71.86 | 4.34 | 14153 |  |  |
| 2021 | 10 |  | 2.53 | 15500 | 71.86 | 3.84 | 15500 |  |  |
| 2021 | 11 |  | 3.30 | 25250 | 71.86 | 4.67 | 25167 | 0.64 | 83 |
| 2021 | 12 |  | 3.74 | 17208 | 60.05 | 4.84 | 16958 | 1.61 | 250 |
| 2022 | 1 |  | 11.87 | 92764 | 19.73 | 15.03 | 89653 | 5.79 | 3111 |
| 2022 | 2 |  | 14.40 | 97417 | 22.03 | 17.03 | 89972 | 9.32 | 7444 |
| 2022 | 3 |  | 22.42 | 199667 | 21.96 | 28.55 | 192694 | 10.61 | 6972 |
| 2022 | 4 |  | 23.96 | 256528 | 22.03 | 28.71 | 244125 | 14.79 | 12403 |
| 2022 | 5 |  | 22.97 | 186958 | 22.03 | 28.38 | 175736 | 12.54 | 11222 |
| 2022 | 6 |  | 28.13 | 319889 | 22.03 | 34.06 | 306361 | 16.72 | 13528 |
| 2022 | 7 |  | 27.36 | 253764 | 22.03 | 32.22 | 233986 | 18.01 | 19778 |
| 2022 | 8 |  | 32.97 | 435736 | 22.03 | 39.90 | 413153 | 19.61 | 22583 |
| 2022 | 9 |  | 30.11 | 500181 | 22.03 | 36.56 | 473444 | 17.68 | 26736 |
| 2022 | 10 |  | 25.71 | 372208 | 22.03 | 30.88 | 351000 | 15.76 | 21208 |
| 2022 | 11 |  | 28.79 | 508458 | 22.03 | 34.89 | 478847 | 17.04 | 29611 |
| 2022 | 12 |  | 26.15 | 433125 | 22.03 | 32.05 | 405944 | 14.79 | 27181 |
| 2023 | 1 |  | 25.27 | 421819 | 22.03 | 30.88 | 391028 | 14.47 | 30792 |
| 2023 | 2 |  | 30.22 | 539944 | 22.03 | 37.06 | 508028 | 17.04 | 31917 |
| 2023 | 3 |  | 33.08 | 709556 | 22.03 | 41.24 | 664611 | 17.36 | 44944 |
| 2023 | 4 |  | 37.25 | 834125 | 22.03 | 44.57 | 781597 | 23.15 | 52528 |
| 2023 | 5 |  | 35.49 | 812306 | 22.03 | 40.90 | 761222 | 25.08 | 51083 |
| 2023 | 6 |  | 37.58 | 930903 | 22.03 | 44.57 | 875278 | 24.12 | 55625 |
| 2023 | 7 |  | 36.48 | 956736 | 22.03 | 42.57 | 895667 | 24.76 | 61069 |
| 2023 | 8 |  | 35.16 | 865903 | 22.03 | 41.74 | 821458 | 22.51 | 44444 |
| 2023 | 9 |  | 35.93 | 1074000 | 22.03 | 42.40 | 1012000 | 23.47 | 62000 |
| 2023 | 10 |  | 34.95 | 941194 | 22.03 | 41.07 | 886236 | 23.15 | 54958 |
| 2023 | 11 |  | 36.26 | 1173194 | 22.03 | 41.24 | 1098861 | 26.69 | 74333 |
| 2023 | 12 |  | 35.71 | 1190639 | 21.89 | 42.40 | 1110847 | 22.83 | 79792 |

Note: CNY: China Yuan
