# Supplementary material for: The impact of the national reimbursement drug list negotiation policy on the accessibility and utilization of evolocumab and alirocumab in different levels of hospitals: an interrupted time series analysis
Source: Front Pharmacol. 2025 Sep 22;16:1612921. doi: 10.3389/fphar.2025.1612921 (PMC12497621; doi:10.3389/fphar.2025.1612921)
Supplement: Supplementary file 3 [file Table3.docx]

**Supplementary Table 3. Comparison of NMPA-Approved Indications versus International Indications for Evolocumab and Alirocumab**

| ****Aspect**** | ****NMPA-Approved Indications (China Label)**** | ****Internationally Approved Indications (FDA/EMA Labels)**** |
| --- | --- | --- |
| **Evolocumab** |  |  |
| **Atherosclerotic Cardiovascular Disease (ASCVD)** | **Secondary prevention in adults with ASCVD**: Reduce risk of MI, stroke, and coronary revascularization. | **Secondary prevention in adults with ASCVD**: Reduce risk of MI, stroke, and coronary revascularization. (Aligned with China) |
| **Primary Hypercholesterolemia (incl. HeFH)** | **Primary hypercholesterolemia (including HeFH) in adults**: Combined with statins and/or other lipid-lowering agents, or as monotherapy if statins are intolerant/contraindicated. | **Primary hypercholesterolemia (including HeFH) in adults**: Combined with statins and/or other lipid-lowering agents, or as monotherapy if statins are intolerant/contraindicated. (Largely consistent with China) |
| **Homozygous Familial Hypercholesterolemia (HoFH)** | **HoFH in adults and adolescents aged 12 and above**: In combination with other lipid-lowering therapies. | **HoFH in adults and adolescents aged 10 and above** (FDA): In combination with other lipid-lowering therapies. **HoFH in adults and adolescents aged 12 and above** (EMA): Combined with other lipid-lowering therapies. (**Difference**: FDA approves for ages ≥10, while EMA aligns with China for ages ≥12) |
| **LDL-C Targets/Thresholds** | Generally described as "reducing LDL-C" without specific thresholds. | More specific language may be used, e.g., FDA approval for **reducing LDL-C in HeFH patients requiring apheresis**. |
| **Other** | / | **Primary prevention of cardiovascular events**: FDA approval for **risk reduction in adults with established CVD**, while EMA wording focuses more on secondary prevention. |
| **Alirocumab** |  |  |
| **CV Risk Reduction** | **Secondary prevention in adults with atherosclerotic cardiovascular disease (ASCVD)**: Reduce risk of MI, stroke, and unstable angina requiring hospitalization. | **Secondary prevention in adults with ASCVD**: Reduce risk of MI, stroke, and unstable angina requiring hospitalization. (Consistent with China) |
| **Primary Hyperlipidemia (incl. HeFH)** | **Primary hypercholesterolemia (including heterozygous familial hypercholesterolemia [HeFH]) in adults**: Combined with statins when LDL-C is uncontrolled despite maximally tolerated statin therapy. | **Primary hypercholesterolemia (including HeFH) in adults**: Adjunct to diet and statin therapy, **or as monotherapy if statin-intolerant or contraindicated**. (**Key difference**: Explicit inclusion of monotherapy) |
| **HoFH** | **Homozygous familial hypercholesterolemia (HoFH) in adults and adolescents aged 12 and above**: In combination with other lipid-lowering therapies. | **HoFH in adults** (FDA): In combination with other lipid-lowering therapies. **HoFH in adults and adolescents aged 12 and above** (EMA): Combined with other lipid-lowering therapies. (**Difference**: FDA approval is for adults only, while EMA aligns with China) |
| **LDL-C Thresholds/Specifics** | Described in general terms. | FDA label specifically includes use **to reduce LDL-C in HeFH patients requiring apheresis**. |
| **Other** | / | / |
